# Supplementary material for: High-throughput ab initio design of atomic interfaces using InterMatch
Source: Nat Commun. 2023 Dec 1;14:7921. doi: 10.1038/s41467-023-43496-5 (PMC10692083; doi:10.1038/s41467-023-43496-5)
Supplement: Supplementary file 1 — Supplementary Information [file 41467_2023_43496_MOESM1_ESM.pdf]

# Supplemental Information: High-Throughput *Ab Initio* Design of Atomic Interfaces using InterMatch

Eli Gerber,[1] Steven B. Torrisi,[2,3] Sara Shabani,[4] Eric Seewald,[4] Jordan Pack,[4] Jennifer E. Hoffman,[2,5] Cory R. Dean,[4] Abhay N. Pasupathy,[4] and Eun-Ah Kim[6]

[1]*School of Applied and Engineering Physics, Cornell University, Ithaca, NY 14853, USA*
[2]*Department of Physics, Harvard University, Cambridge, MA 02138, USA*
[3]*Energy & Materials Division, Toyota Research Institute, Los Altos, CA 94022, USA*
[4]*Department of Physics, Columbia University, New York, NY, USA*
[5]*John A. Paulson School of Engineering and Applied Sciences, Harvard University, Cambridge, MA 02138, USA*
[6]*Department of Physics, Cornell University, Ithaca, NY 14853, USA*

(Dated: October 22, 2023 [file: output])

In Section I we present additional InterMatch results identifying optimal substrate candidates for forming high-charge-transfer interfaces with group-VI transition metal dichalcogenides and putative spin liquid material $\alpha$-$RuCl_3$. In Section II we provide DFT verification of interface lattice constants and interlayer separation distances for all systems shown in Figure 3(c) of the main text. In Section III we illustrate the effects of including different physical parameters in the GR/$\alpha$-$RuCl_3$ superlattice search criteria.

## I. SUPPLEMENTARY INFORMATION I: CHARGE TRANSFER SUBSTRATES FOR $WTe_2$, $WSe_2$, AND $\alpha$-$RuCl_3$

We further demonstrate InterMatch by applying it to two types of materials: group-VI TMDs, and putative spin liquid $\alpha$-$RuCl_3$[1]. TMDs' unique combination of properties makes them highly attractive for nanoelectronics applications and fundamental studies of novel physical phenomena[2]. However, the realization of many such high-performance devices and exotic phases is limited by the availability of systems with high carrier mobility and low contact resistances between metal contacts and the semiconductor. Quantum spin liquids (QSLs) are interacting quantum systems in which spins do not order at low temperatures, and have been theorized to offer insights into high-temperature superconductivity upon doping. $\alpha$-$RuCl_3$, for example, has been intensively discussed as a possible candidate for Kitaev physics; however, it orders antiferromagnetically at low temperatures due to the presence of additional magnetic couplings extending beyond the pure Kitaev interaction. Doping $\alpha$-$RuCl_3$ with charge carriers has been predicted to enhance Kitaev interactions and push $\alpha$-$RuCl_3$ closer to the spin liquid phase. We use InterMatch to identify stable, high-charge-transfer interfaces for electron- and hole-doping the TMDs $WTe_2$, $WSe_2$, and $MoSe_2$, and the putative QSL $\alpha$-$RuCl_3$. Fig. SMS1 shows sample InterMatch results of substrate candidates for interfaces with $WTe_2$, $WSe_2$, and $\alpha$-$RuCl_3$, highlighting those that minimize elastic energy $E_{el}$ and maximize charge transfer $\Delta n$. In Fig. SMS1 (a)-(b), we survey all $\sim 70,000$ oxides from the Materials Project. Oxides have a wide range of charge neutrality levels and therefore constitute a powerful addition to electrostatic gating or chemical doping for controlling carrier concentration in heterostructures. In Fig. SMS1 (c) we survey all entries in the 2DMatpedia database, and determine two-dimensional (2D) substrate candidates to maximally dope $\alpha$-$RuCl_3$.

## II. SUPPLEMENTARY INFORMATION II: COMPARISON OF INTERMATCH PREDICTIONS WITH DFT: LATTICE CONSTANTS AND INTERLAYER SEPARATION DISTANCE

We provide further verification of InterMatch predictions for the $MoSe_2$ interfaces in Fig. 2 (c)-(d) of the main text, by comparing the relaxed interface lattice constants as well as the interlayer separation distances for each system predicted by InterMatch with those obtained via supercell DFT. The results are shown in Fig. S2.

## III. SUPPLEMENTARY INFORMATION III: GR/$\alpha$-$RuCl_3$ MOIRÉ PATTERNS

In this section we illustrate by means of concrete examples how different constraints affect superlattice predictions and allow for the prediction of longer moiré periods. For reference, we compute the series of parametric period/angle curves of GR/$\alpha$-$RuCl_3$ moirés using the model from Ref[3], in which only GR is allowed to be strained, and only isotropic compression/expansion of the GR layer is permitted. These curves are shown in Figure S3 (a). We then run an InterMatch superlattice search with the same constraints, allowing only isotropic strain in the GR layer. These

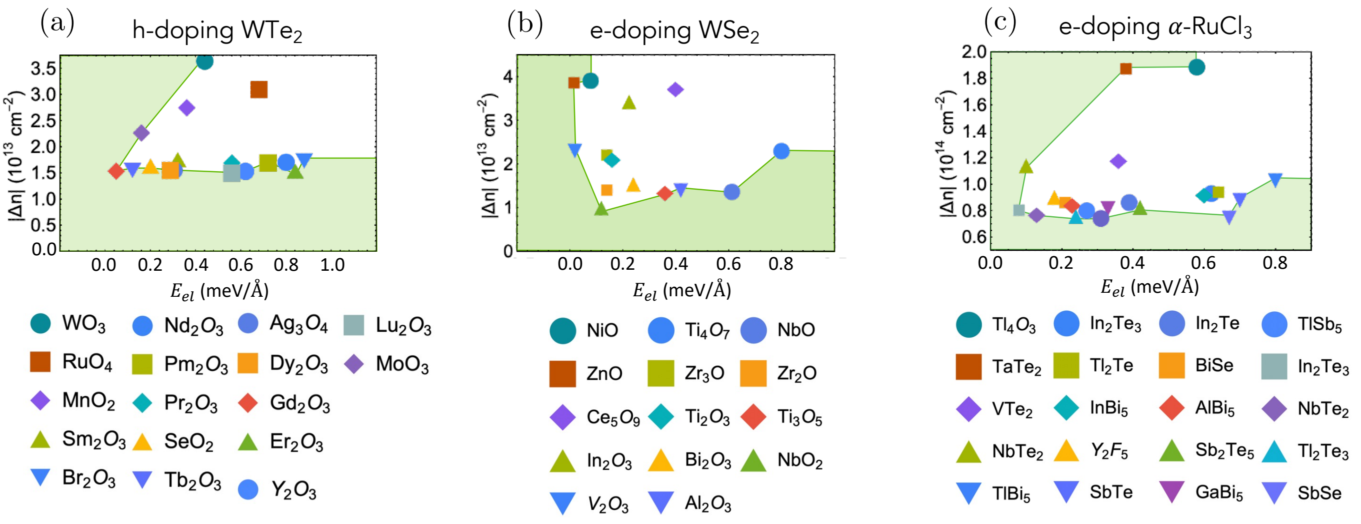


FIG. S1: Sample InterMatch results for different charge transfer interfaces. The solid green line indicates the pareto-optimal frontier of substrate candidates that minimize elastic energy $E_{el}$ and maximize charge transfer $|\Delta n|$. Calculated quantities in (a)-(b) use *ab initio* data from the Materials Project as inputs, while those in (c) use data from the 2DMatpedia database. Source data are provided as a Source Data file.

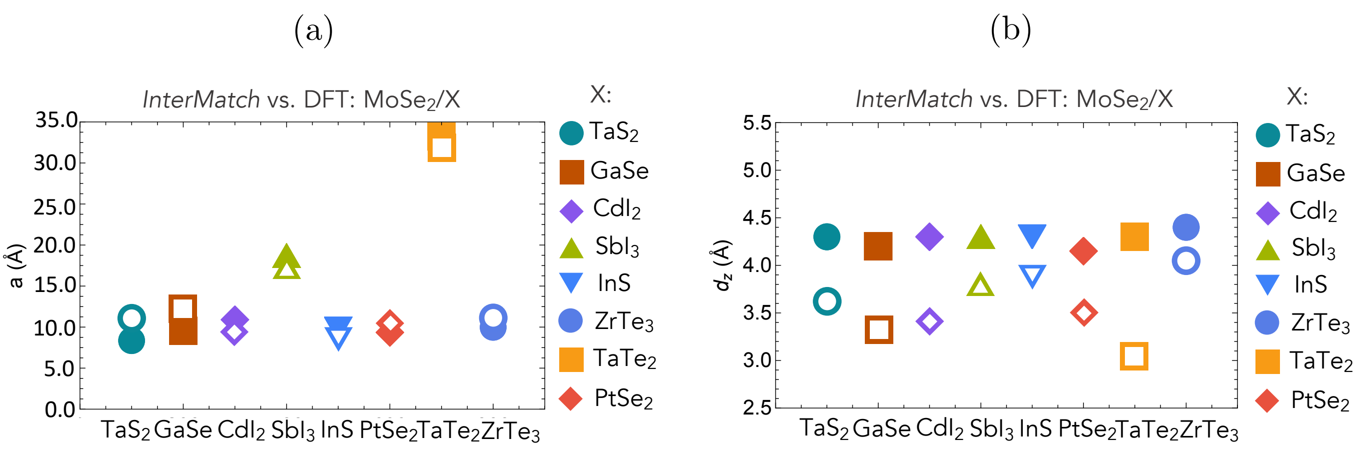


FIG. S2: Additional benchmarking of InterMatch. (a) Interface lattice constants predicted by InterMatch (solid symbols) vs DFT (open symbols). (b) Interlayer separation distances of interfaces predicted by InterMatch (solid symbols) vs DFT (open symbols). Source data are provided as a Source Data file.

superlattices are represented by points overlaid on the parametric curves in Figure S3 (a). Under these constraints, both calculations predict moiré length scales only on the order of the shortest ones observed in the experiment to be stable.

We then introduce (i) anisotropic strains in either layer (ii) elastic energy as a stability criterion and (iii) small ($\pm 1^\circ$) deviations in the moiré angle $\theta_m$ from $60^\circ$. The lattices predicted under these constraints are shown in Figure S3 (b). Below we provide examples of how each of the conditions (i)-(iii) can allow for much larger moiré periods to be predicted.

i First, consider an example of GR/$\alpha$-$RuCl_3$ superlattices at a twist angle of $\theta = 19^\circ$. Allowing only isotropic strain in GR and using only this % strain as a selection criterion, the longest stable superlattice period predicted is $L =$ 2.6 nm (square in Figure S3 (a)) with lattice coefficients $(i_{11}, j_{11}, i_{21}, j_{21}, i_{12}.j_{12}, i_{22}, j_{22}) = (10, 11, 11, 1, 3, 5, 5, 2)$. This configuration requires $\varepsilon_{11} = \varepsilon_{22} = -0.8\%$ strain to become commensurate. If we now allow for small ($< 1\%^4$) anisotropic strains, we find an additional stable superlattice at $\theta = 19^\circ$ with period $L = 13.6$ nm

(square in Figure S3 (b)) and lattice coefficients $(i_{11}, j_{11}, i_{21}, j_{21}, i_{12}.j_{12}, i_{22}, j_{22}) = (40, 63, 63, 23, 9, 26, 26, 17)$. This configuration requires only 0.2% strain in $\varepsilon_{11}$ and 0.3% strain in $\varepsilon_{22}$ and is favored even from the perspective of % strain alone.

ii Second, consider an example at a twist angle of $\theta = 16°$. Allowing only isotropic strain in GR and using only the strain as a selection criterion, the largest superlattice period predicted is $L = 3.7$ nm (star in Figure S3 (a)) with lattice coefficients $(i_{11}, j_{11}, i_{21}, j_{21}, i_{12}.j_{12}, i_{22}, j_{22}) = (0, 15, 15, 15, -2, 5, 5, 7)$. This configuration requires $\varepsilon_{11} = \varepsilon_{22} = 0.05\%$ strain to become commensurate. If we allow anisotropic strains and allow $\alpha$-$RuCl_3$ to be strained as well, there is an additional near-commensurate superlattice at $\theta = 16°$ with a much larger period of $L = 20$ nm (star in Figure S3 (b)) and lattice coefficients $(i_{11}, j_{11}, i_{21}, j_{21}, i_{12}.j_{12}, i_{22}, j_{22}) = (51, 97, 94, 50, 11, 39, 37, 29)$. This configuration requires $\varepsilon_{12} = 0.17\%$ shear strain to become commensurate. Clearly this second configuration is not favored from the perspective of total % strain, however, if we now introduce elastic energy $E_{el} = \frac{1}{2}C_{ijkl}\varepsilon_{ij}\varepsilon_{kl}$ as a lattice selection criterion, computing $E_{el}$ for each we find that for the first superlattice $E_{el} = \frac{1}{2}(2 \times 904 \text{ GPa})(5 \times 10^{-4})^2 = 1.41 \times 10^{-5}$ eV/Å$^3$ while for the second superlattice $E_{el} = \frac{1}{2}(15.8 \text{ GPa})(17 \times 10^{-4})^2 = 1.42 \times 10^{-6}$ eV/Å$^3$. Here we have used the elastic constants reported for $\alpha$-$RuCl_3$[5] and GR[6].

iii Next, consider an example at a twist angle of $\theta = 14°$. With only isotropic GR strains allowed, the largest superlattice period predicted is $L = 3.9$ nm (triangle in Figure S3 (a)) with lattice coefficients $(i_{11}, j_{11}, i_{21}, j_{21}, i_{12}.j_{12}, i_{22}, j_{22}) = (-15, 2, 2, 17, -7, -1, -1, 6)$, requiring $\varepsilon_{11} = \varepsilon_{22} = -1.5\%$ strain to become commensurate. If we now allow for small strain anisotropy and slightly relax the constraints on $\theta_m$ so that $\theta_m$ can vary by $\pm 1°$, we find an additional superlattice at $\theta = 14°$ with period $L = 21$ nm (triangle in Figure S3 (b)), lattice coefficients $(i_{11}, j_{11}, i_{21}, j_{21}, i_{12}.j_{12}, i_{22}, j_{22}) = (51, 99, 100, 51, 12, 40, 40, 29)$, and $\theta_m = 59°$. This configuration requires only $\varepsilon_{11} = 0.2\%$ and $\varepsilon_{22} = -0.19\%$ strain to become commensurate and is favored both from the perspective of strain and elastic energy.

iv Finally, consider an example at a twist angle of $\theta = 12°$. With only isotropic GR strains allowed, the largest superlattice period predicted is 6.1 nm (diamond in Figure S3 (a)) with lattice coefficients $(i_{11}, j_{11}, i_{21}, j_{21}, i_{12}.j_{12}, i_{22}, j_{22}) = (1, 25, 25, 24, -2, 9, 9, 11)$, requiring $\varepsilon_{11} = \varepsilon_{22} = -0.02\%$ strain to become commensurate. If we now allow for conditions (i)-(iii), that is, anisotropic strains, elastic energy, and $59° \leq \theta_m \leq 61°$, we find an additional superlattice at $\theta = 12°$ with period $L = 25.8$ nm (diamond in Figure S3 (b)), lattice coefficients $(i_{11}, j_{11}, i_{21}, j_{21}, i_{12}.j_{12}, i_{22}, j_{22}) = (61, 118, 119, 62, 16, 48, 48, 34)$, and $\theta_m = 59.4°$. This configuration requires $-0.012\%$ strain in $\varepsilon_{11}$ and 0.018% strain in $\varepsilon_{22}$ and is favored from the perspective of strain and elastic energy.

In all examples above, the $i_{kl}$ are integer coefficients for system $i$ making up the $2 \times 2$ matrices $\mathbf{M}^i$ defined in Equation (2) of the main text.

## IV. SUPPLEMENTARY REFERENCES

[1] K. W. Plumb, J. P. Clancy, L. J. Sandilands, V. V. Shankar, Y. F. Hu, K. S. Burch, H.-Y. Kee, and Y.-J. Kim, Physical Review B **90**, 041112 (2014).
[2] S. Manzeli, D. Ovchinnikov, D. Pasquier, O. V. Yazyev, and A. Kis, Nature Reviews Materials **2**, 17033 (2017).
[3] A. Artaud, L. Magaud, T. Le Quang, V. Guisset, P. David, C. Chapelier, and J. Coraux, Scientific Reports **6**, 25670 (2016).
[4] Y. Choi, J. Kemmer, Y. Peng, A. Thomson, H. Arora, R. Polski, Y. Zhang, H. Ren, J. Alicea, G. Refael, et al., Nature Physics **15**, 1174 (2019).
[5] E. Deligoz, H. Bogaz Özişik, and H. Ozisik, The International Congress on The World of Technology and Advanced Materials (2018).
[6] A. Jain, S. P. Ong, G. Hautier, W. Chen, W. D. Richards, S. Dacek, S. Cholia, D. Gunter, D. Skinner, G. Ceder, et al., APL Materials **1**, 011002 (2013).

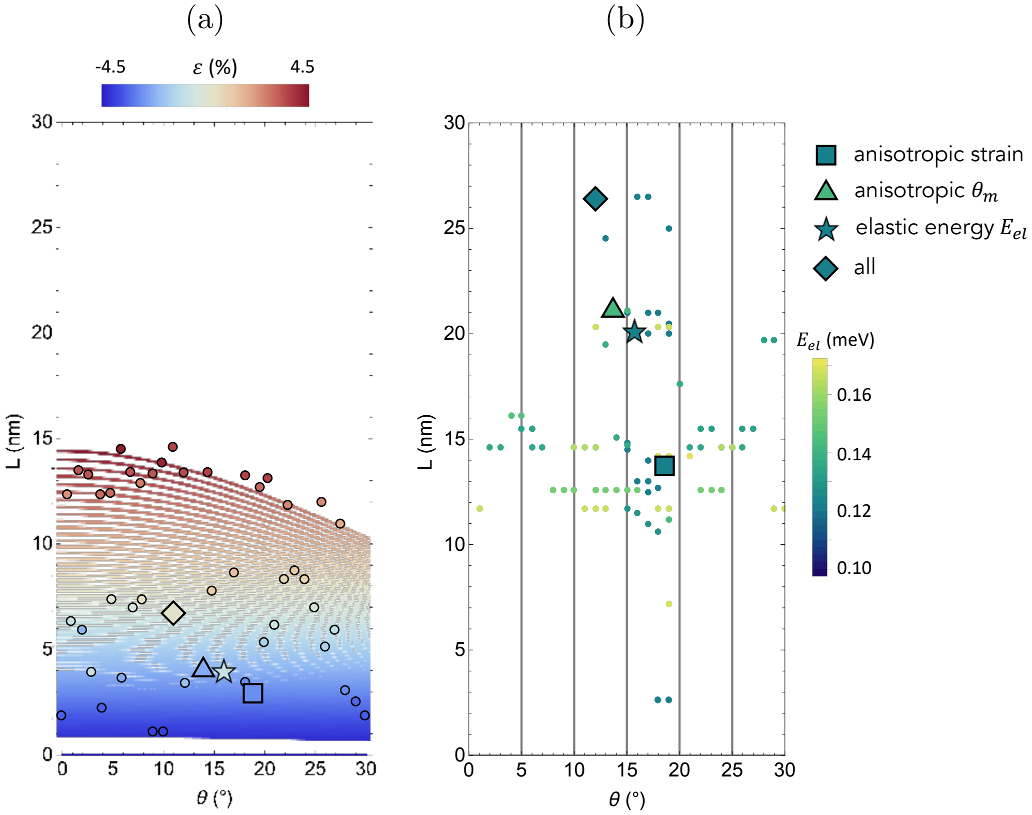


FIG. S3: Predicted GR/$\alpha$-$RuCl_3$ superlattices using different models and criteria. (a) Parametric period/angle curves with isotropic strain $\varepsilon$ in GR according to the model in Ref[3]. Overlaid are points denoting InterMatch search results for superlattices having only isotropic strain in GR with $\theta_m = 60°$. (b) InterMatch results including anisotropic strain, elastic energy $E_{el}$, and $59° \leq \theta_m \leq 61°$. Enlarged plot markers (square, triangle, star, diamond) correspond to search criteria that can lead to longer superlattice periods observed in experiment. Source data are provided as a Source Data file.
